# Long-term efficacy and safety of ravulizumab in adults with anti-acetylcholine receptor antibody-positive generalized myasthenia gravis: results from the phase 3 CHAMPION MG open-label extension

**DOI:** 10.1007/s00415-023-11699-x

**Published:** 2023-04-27

**Authors:** Andreas Meisel, Djillali Annane, Tuan Vu, Renato Mantegazza, Masahisa Katsuno, Rasha Aguzzi, Glen Frick, Laura Gault, James F. Howard, the CHAMPION MG Study Group

**Affiliations:** 1 Charité Universitätsmedizin Berlin, Berlin, Germany; 2 Hôpital Raymond Poincaré, Garches, France; 3 University of South Florida Morsani College of Medicine, Tampa, FL USA; 4 Fondazione IRCCS Istituto Neurologico Carlo Besta, Milan, Italy; 5 Nagoya University Graduate School of Medicine, Nagoya, Japan; 6 Alexion, AstraZeneca Rare Disease, Boston, MA USA; 7 The University of North Carolina, Chapel Hill, NC USA

**Keywords:** Ravulizumab, Generalized myasthenia gravis, Open-label, Long-term

## Abstract

**Introduction:**

Ravulizumab demonstrated efficacy and an acceptable safety profile versus placebo in the randomized controlled period (RCP) of the phase 3 CHAMPION MG trial in patients with anti-acetylcholine receptor antibody-positive generalized myasthenia gravis. We report an interim analysis of the ongoing open-label extension (OLE) designed to evaluate long-term treatment effects.

**Methods:**

Following completion of the 26-week RCP, patients could enter the OLE; patients who received ravulizumab in the RCP continued the drug; patients who previously received placebo switched to ravulizumab. Patients receive body-weight-based maintenance dosing of ravulizumab every 8 weeks. Efficacy endpoints up to 60 weeks included Myasthenia Gravis–Activities of Daily Living (MG-ADL) and Quantitative Myasthenia Gravis (QMG) scores, with least-squares (LS) mean change and 95% confidence intervals (95% CI) reported.

**Results:**

Long-term efficacy and safety in the OLE were analyzed in 161 and 169 patients, respectively. Improvements in all scores were maintained through 60 weeks in patients who received ravulizumab during the RCP; LS mean change from RCP baseline in MG-ADL score was − 4.0 (95% CI: − 4.8, − 3.1; p < 0.0001). Rapid (within 2 weeks) and sustained improvements occurred in patients previously receiving placebo; LS mean change in MG-ADL score from OLE baseline to Week 60 was − 1.7 (95% CI: − 2.7, − 0.8; p = 0.0007). Similar trends were seen in QMG scores. Ravulizumab treatment was associated with a decreased rate of clinical deterioration events compared with placebo. Ravulizumab was well tolerated; no meningococcal infections were reported.

**Conclusion:**

Findings support the sustained efficacy and long-term safety of ravulizumab, administered every 8 weeks, in adults with anti-acetylcholine receptor antibody-positive generalized myasthenia gravis.

ClinicalTrials.gov identifier: NCT03920293; EudraCT: 2018-003243-39.

**Supplementary Information:**

The online version contains supplementary material available at 10.1007/s00415-023-11699-x.

## Introduction

Myasthenia gravis (MG) is a rare, chronic, autoimmune condition that affects the neuromuscular junction [1, 2]. It is characterized by fatigable muscle weakness that generally worsens with activity and improves with rest [1, 2]. The most common initial presentation is ocular weakness, but progression to generalized MG (gMG) involving muscles of the head, neck, trunk, limbs, and/or respiratory system occurs within 2 years of disease onset in up to 88% of patients [3, 4]. The burden of MG is substantial, with many patients reporting major impacts on daily living and quality of life [5–8]. Approximately 15% of patients with MG experience myasthenic crisis, characterized by respiratory failure and requiring mechanical ventilation [9].

Approximately 85% of patients with gMG have autoantibodies directed against the acetylcholine receptor (AChR) [10–12]. Complement activation is a key pathologic mechanism in patients who are anti-AChR antibody-positive [10–12]. Binding of autoantibodies to the AChR has been shown to lead to activation of the complement cascade and formation of the complement membrane attack complex, which results in architectural destruction of the neuromuscular junction post-synaptic membrane and compromised neuromuscular transmission [10–14].

Traditional therapies for MG include oral cholinesterase inhibitors and long-term immune therapies, such as corticosteroids and non-steroidal immunosuppressants (including azathioprine, mycophenolate mofetil, cyclosporin, and tacrolimus) [15]. Long-term immune therapies are based on non-specific immunosuppression and are associated with significant side effects that can impact daily life [16–18]. Inhibiting the complement pathway offers a more targeted approach to therapy [16, 17]. Eculizumab—a humanized monoclonal antibody that binds specifically and with high affinity to terminal complement protein C5—was the first human C5 inhibitor to be approved for the treatment of anti-AChR antibody-positive gMG. Efficacy and safety of eculizumab administered every 2 weeks was demonstrated in a phase 3 study in adults with anti-AChR antibody-positive refractory gMG [19, 20]. Ravulizumab was engineered from eculizumab via four selected amino acid substitutions to maintain therapeutic serum concentrations over an 8-week dosing interval [21]. These substitutions result in reduced target-mediated drug disposition (by increasing dissociation of the antibody from C5 in the endosome) and enhanced neonatal Fc receptor-mediated recycling of the unbound antibody, thereby extending the molecule’s elimination half-life and its duration of action [21]. Ravulizumab is approved for use in patients with paroxysmal nocturnal hemoglobinuria (PNH), atypical hemolytic uremic syndrome (aHUS), or anti-AChR antibody-positive gMG, and has been shown to provide complete terminal C5 complement inhibition throughout an 8-week dosing interval in these conditions [22–24]. Further investigation in patients with PNH showed that serum IgG concentrations, which are regulated by the neonatal Fc receptor, were not affected by ravulizumab treatment [25].

We have previously reported data from the phase 3, randomized, placebo-controlled CHAMPION MG study showing that ravulizumab is well tolerated and improves clinical outcomes compared with placebo in patients with anti-AChR antibody-positive gMG [26]. Here, we report results from a prespecified interim analysis of the ongoing open-label extension (OLE) of CHAMPION MG, including data for up to 60 weeks of ravulizumab treatment.

## Methods

### Study design and patients

**Fig. 1:**
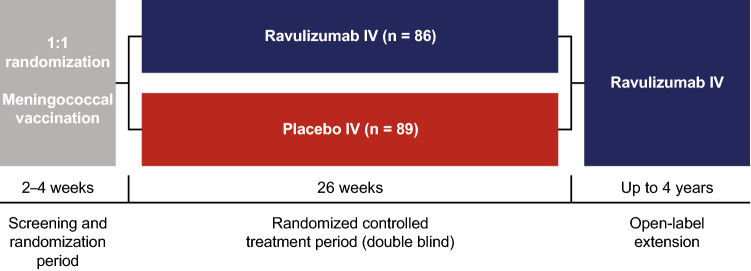
Study design. IV, intravenous. From NEJM Evidence, Vu T, et al, Terminal complement inhibitor ravulizumab in generalized myasthenia gravis, 1, EvidDoa2100066. Copyright © 2022 Massachusetts Medical Society. Reprinted with permission from Massachusetts Medical Society

The CHAMPION MG study consists of a 26-week, double-blind, randomized, placebo-controlled period (RCP), followed by an ongoing OLE of up to 4 years (Fig. 1). The methodology of the RCP has been previously published [26] and is described in brief here. The study was conducted in accordance with the International Conference on Harmonisation Guideline for Good Clinical Practice and the Declaration of Helsinki. Institutional review board or independent ethics committee approval was obtained and patients gave written informed consent. The study is registered with ClinicalTrials.gov (NCT03920293) and EudraCT (2018-003243-39).

Adult patients (≥ 18 years of age), diagnosed with MG ≥ 6 months before screening and who were anti-AChR antibody-positive, were included in the study. Additionally, patients had to have Myasthenia Gravis Foundation of America class II–IV disease and a Myasthenia Gravis–Activities of Daily Living (MG-ADL) total score of ≥ 6 at screening and randomization. As patients treated with C5 inhibitors are at increased risk of meningococcal infection, all participants were required to be vaccinated against *Neisseria meningitidis* within 3 years before starting trial agents; those who initiated a trial agent < 2 weeks after receiving a meningococcal vaccine received appropriate prophylactic antibiotics until 2 weeks after vaccination. Patients were excluded if they had received intravenous immunoglobulin or plasma exchange in the previous 4 weeks, rituximab treatment in the previous 6 months, or previous treatment with complement inhibitors (e.g. eculizumab).

### Treatments

During the RCP, patients were randomized 1:1 to intravenous ravulizumab or matching placebo. Ravulizumab dosing during the study was based on patients’ body weight. In the RCP, patients received an initial loading dose of 2400, 2700, or 3000 mg on Day 1, followed by a maintenance dose of 3000, 3300, or 3600 mg starting on Day 15 and every 8 weeks thereafter. On completing the RCP, patients were invited to enter the OLE, which started for each patient when he or she received a dose of ravulizumab at Week 26 of the study. The doses and dosing schedule at the start of the OLE were designed to ensure that patients and study personnel remained blinded to the treatment assignment in the RCP. Therefore, at Week 26, patients who had received ravulizumab during the RCP (ravulizumab–ravulizumab group) received ravulizumab 900 mg, and patients who had received placebo (placebo–ravulizumab group) received a body-weight-based loading dose of 2400, 2700, or 3000 mg. For the next scheduled maintenance dose at Week 28 and every 8 weeks thereafter for up to 4 years, all patients received body-weight-based doses of 3000, 3300, or 3600 mg.

Patients receiving stable therapy with cholinesterase inhibitors and immunosuppressants at the start of the study could continue them. Dose changes were not permitted during the RCP, but were allowed during the OLE at the investigator’s discretion. Rescue therapy, including high-dose corticosteroids, plasma exchange, or intravenous immunoglobulin, was permitted throughout the study for patients experiencing clinical deterioration.

### Study endpoints

Efficacy was evaluated using the following validated measures: the MG-ADL score [27]; Quantitative Myasthenia Gravis (QMG) score [28]; revised 15-item Myasthenia Gravis Quality of Life (MG-QOL15r) score [29]; and Neurological Quality of Life (Neuro-QoL) Fatigue subscale score [30, 31].

The MG-ADL scale is an 8-item survey of patient-reported MG symptom severity with a total score range of 0–24. The QMG is a 13-item clinician assessment of strength with a total score range of 0–39. The MG-QOL15r is a 15-item questionnaire on health-related quality of life with a total score range of 0–30. The Neuro-QoL Fatigue subscale evaluates the effect of fatigue on the quality of life of patients with neurologic disorders. The long form of the subscale used in this study comprises 19 patient-reported items with a total score range of 19–95. For each of the four measures, a reduction in scores indicates an improvement.

MG-ADL and QMG were assessed at screening, baseline, and Weeks 1, 2, 4, 10, 12, 18, and 26 of the RCP, and at Weeks 28, 30, 36, 38, 44, 52, and 60 during the OLE. MG-QOL15r and Neuro-QoL Fatigue subscale scores were assessed at baseline and Weeks 4, 12, 18, and 26 of the RCP, and at Weeks 30, 38, 44, 52, and 60 during the OLE. At each assessment, the order of endpoints assessed was MG-ADL, QMG, MG-QOL15r, and Neuro-QoL Fatigue. Administration of the MG-ADL questionnaire and QMG and MG-QOL15r assessments was performed by trained clinical evaluators; preferably the same person evaluated each patient throughout the study. Assessment of QMG was performed at approximately the same time of day at each scheduled visit if possible. Patients who were receiving a cholinesterase inhibitor during the RCP or OLE were required to refrain from taking it for at least 10 h before each scheduled assessment.

Study endpoints were change from RCP or OLE baseline in MG-ADL, QMG, MG-QOL15r, and Neuro-QoL Fatigue scores. The primary endpoint was change in MG-ADL score at Week 60. In addition, a responder analysis of MG-ADL and QMG scores was performed, with a clinical response defined as an improvement from baseline of ≥ 3 points for MG-ADL or ≥ 5 points for QMG. Occurrence of clinical deterioration and the use of rescue therapy by patients experiencing clinical deterioration were also assessed. Clinical deterioration was defined as: an MG crisis (weakness severe enough to necessitate intubation or to delay extubation following surgery); significant symptomatic worsening (worsening to a score of 3, or a 2-point worsening from baseline, on any of the individual MG-ADL items other than double vision or eyelid droop, which in the investigator’s assessment was associated with significant symptomatic worsening); or use of rescue therapy for health in jeopardy (administration of rescue therapy to a patient whose health, in the opinion of the investigator, would be in jeopardy if rescue therapy were not given).

Efficacy data were analyzed as follows: change from RCP baseline at Week 60 was assessed for all endpoints in both study groups (ravulizumab–ravulizumab and placebo–ravulizumab); change from OLE baseline to OLE Week 2 (MG-ADL and QMG scores), OLE Week 4 (MG-QOL15r and Neuro-QoL Fatigue scores), and OLE Week 34 (all endpoints) was assessed for the placebo–ravulizumab group only. The analysis from the RCP baseline allowed assessment of endpoints throughout the entire study, while the analysis from the OLE baseline allowed assessment of the immediate and longer-term impact of switching to ravulizumab in the placebo–ravulizumab group.

Changes in corticosteroid use during the OLE were also assessed. The safety and tolerability of ravulizumab were evaluated based on the incidence of adverse events, clinical laboratory and vital sign findings, and electrocardiogram abnormalities throughout the study (RCP and OLE periods). The potential relationship between each adverse event and the study agent was evaluated by the principal investigators, who were trained in causality assessment. Serious adverse events were also reviewed by the sponsor.

### Statistical analysis

Sample-size calculations for the RCP have been described previously [26]. Efficacy was assessed in the full analysis set, which included all patients who were randomized to treatment and received at least one dose of study agent, and in the OLE analysis set, which comprised all patients who received at least one dose of ravulizumab in the OLE. Safety was assessed in the safety analysis set, which comprised all patients who received at least one dose of ravulizumab in the RCP or OLE by the time of data cut-off. Adverse events with onset after the first ravulizumab infusion are reported.

A mixed model for repeated measures (MMRM) was used to analyze changes from baseline, with the assumption that missing data were missing at random. Missing data were not imputed. Data are shown as least-squares (LS) mean change from baseline with 95% confidence intervals (CIs). The estimates for the RCP were based on an MMRM that included treatment group, stratification factor region, baseline score, study visit, and study visit by treatment group interaction. Visits up to Week 26 were included in the model. The OLE estimates were based on an MMRM that included stratification factor region, baseline score, and study visit. A model was fit for the ravulizumab–ravulizumab and placebo–ravulizumab arms of the OLE analysis set separately.

The responder analysis calculated the proportion of patients who achieved a clinical response from the RCP baseline in patients who initiated ravulizumab in the RCP and from the OLE baseline in patients who initiated ravulizumab in the OLE.

A generalized estimating equation Poisson regression repeated measures model was used to analyze clinical deterioration event rates, with the number of events as the dependent variable, the logarithm of patient-years as the offset variable, and the study phase indicator (pre-study, placebo, or ravulizumab) as the explanatory factor.

The present interim analysis was prespecified. Data cut-off was 9 November 2021, including data for up to 60 weeks from the RCP baseline (34 weeks from the OLE baseline when patients receiving placebo during the RCP switched to ravulizumab). All analyses were performed using Statistical Analysis Software Version 9.4 (SAS^®^, SAS Institute, Cary, NC, USA)*.*

## Results

**Fig. 2:**
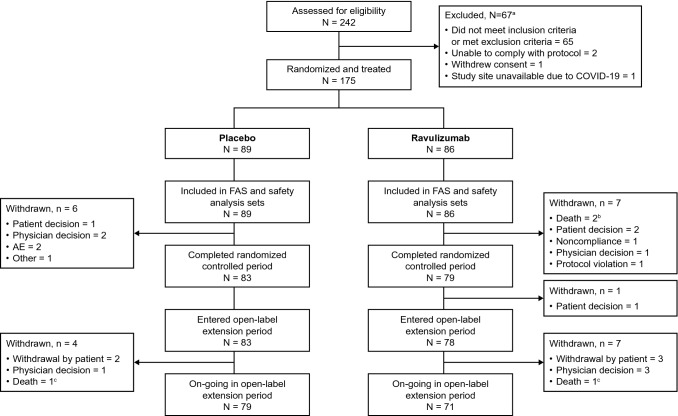
Patient disposition. ^a^Individuals may have had more than one reason for exclusion^. b^One death was due to COVID-19 and one was due to cerebral hemorrhage. ^c^Both deaths were related to COVID-19. AE, adverse event; COVID-19, coronavirus disease 2019; FAS, full analysis set

A total of 175 patients were enrolled into the RCP (full analysis set) between March 2019 and November 2020 across 85 centers in 13 countries; 86 patients received ravulizumab and 89 received placebo. Six patients received prophylactic antibiotics until ≥ 2 weeks after meningococcal vaccination. A total of 162 patients completed the RCP, of whom 161 entered the OLE (OLE analysis set); 78 patients were in the ravulizumab–ravulizumab group and 83 were in the placebo-ravulizumab group (Fig. 2). The safety set comprised 169 patients who received ravulizumab at any point during the RCP or OLE: 86 in the ravulizumab–ravulizumab group who received ravulizumab during the RCP (of whom eight did not continue to the OLE); and 83 who received placebo during the RCP and switched to ravulizumab for the OLE. Eleven patients withdrew from the OLE before data cut-off (Fig. 2). A total of 55 patients in the ravulizumab–ravulizumab group and 58 in the placebo–ravulizumab group completed the Week 60 visit before the data cut-off. All patients had entered the study at least 52 weeks before data cut-off.

Baseline (RCP entry) demographic and clinical characteristics were similar in the two treatment groups who entered the OLE (Table 1). Median duration (range) of follow-up was 442 (243–466) days in the ravulizumab–ravulizumab group and 442 (269–461) days in the placebo–ravulizumab group. Median duration (range) of ravulizumab treatment at the time of data cut-off was 421 (14–442) days in the ravulizumab–ravulizumab group and 239 (63–258) days in the placebo–ravulizumab group.

**Table 1:** Demographic and clinical characteristics at RCP entry of patients included in the OLE analysis set

Characteristic	Ravulizumab–ravulizumab^a^ (n = 78)	Placebo–ravulizumab^b^ (n = 83)	All patients (N = 161)
Female, n (%)	40 (51.3)	42 (50.6)	82 (50.9)
Age, years, mean ± SD	58.2 ± 13.6	53.6 ± 16.4	55.9 ± 15.2
Race, n (%)			
White	61 (78.2)	57 (68.7)	118 (73.3)
Asian	13 (16.7)	14 (16.9)	27 (16.8)
Black or African American	2 (2.6)	4 (4.8)	6 (3.7)
Other/unknown/not reported	2 (2.6)	8 (9.6)	10 (6.2)
MGFA clinical classification, n (%)			
Class IIa/b	36 (46.2)	35 (42.2)	71 (44.1)
Class IIIa/b	37 (47.4)	43 (51.8)	80 (49.7)
Class IVa/b	5 (6.4)	5 (6.0)	10 (6.2)
MG-ADL total score, mean ± SD	9.2 ± 2.6	8.9 ± 2.2	9.0 ± 2.4
QMG total score, mean ± SD	14.8 ± 5.2	14.3 ± 5.2	14.5 ± 5.2

MG-ADL, Myasthenia Gravis–Activities of Daily Living; MGFA, Myasthenia Gravis Foundation of America; OLE, open-label extension; QMG, Quantitative Myasthenia Gravis; RCP, randomized controlled period; SD, standard deviation

^a^Patients treated with ravulizumab during both the RCP and OLE. ^b^Patients treated with placebo during the RCP and ravulizumab during the OLE

### Long-term efficacy

**Fig. 3:**
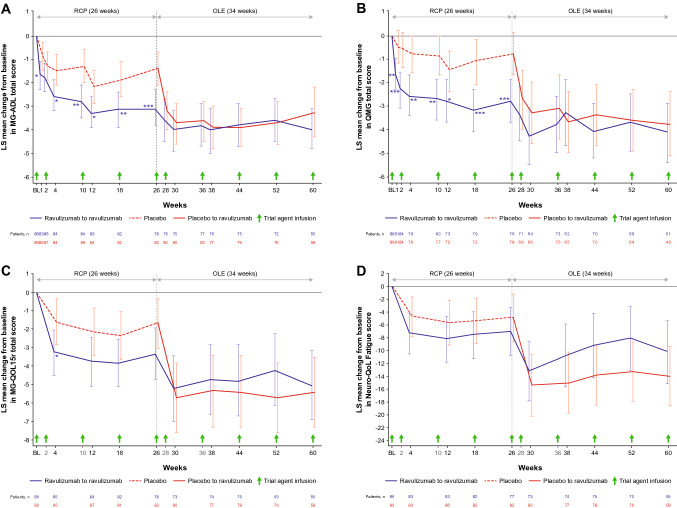
Least-squares mean change (95% CI) from RCP baseline in **A** MG-ADL total score, **B** QMG total score, **C** MG-QOL15r total score and **D** Neuro-QoL Fatigue score. *, **, and *** indicate two-sided p-values of < 0.05, < 0.01, and < 0.001, respectively, for the comparison of treatment groups in change from baseline during the RCP (p-values are nominal for comparisons at all timepoints except Week 26; endpoints at Week 26 were tested in a hierarchical manner). The RCP estimates are based on an MMRM that included treatment group, stratification factor region, baseline score, study visit, and study visit by treatment group interaction. Visits up to Week 26 were included in the model. The OLE estimates are based on an MMRM that included stratification factor region, baseline score, and study visit. A model was fit for the ravulizumab–ravulizumab and placebo–ravulizumab arms of the OLE analysis set separately. Data for the full analysis set are shown for the RCP; data for the OLE analysis set are shown for the OLE period. Data are offset for clarity. BL, baseline; CI, confidence interval; LS, least squares; MG-ADL, Myasthenia Gravis–Activities of Daily Living; MG-QOL15r, revised 15-item Myasthenia Gravis Quality of Life; MMRM, mixed model for repeated measures; Neuro-QoL, Neurological Quality of Life; OLE, open-label extension; QMG, Quantitative Myasthenia Gravis; RCP, randomized controlled period

**Fig. 4:**
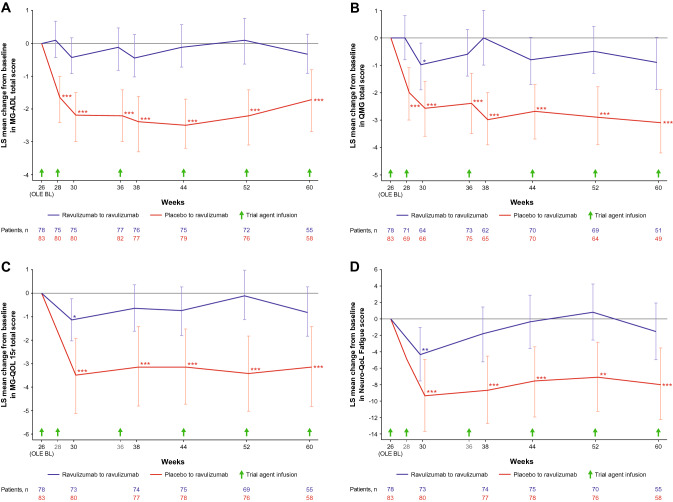
Least-squares mean change (95% CI) from OLE baseline in **A** MG-ADL total score, **B** QMG total score, **C** MG-QOL15r total score and **D** Neuro-QoL Fatigue score. *, **, and *** indicate two-sided nominal p-values of < 0.05, < 0.01, and < 0.001, respectively, for the change from OLE baseline (whether LS mean change equals zero). Estimates were based on an MMRM for each treatment sequence and included stratification factor region, baseline score, and study visit. Data for the OLE analysis set are shown. Data are offset for clarity. BL, baseline; CI, confidence interval; LS, least squares; MG-ADL, Myasthenia Gravis–Activities of Daily Living; MG-QOL15r, revised 15-item Myasthenia Gravis Quality of Life; MMRM, mixed model for repeated measures; Neuro-QoL, Neurological Quality of Life; OLE, open-label extension; QMG, Quantitative Myasthenia Gravis; RCP, randomized controlled period

In the ravulizumab–ravulizumab group, clinical improvements observed during the RCP in the MG-ADL, QMG, MG-QOL15r, and Neuro-QoL Fatigue total scores were sustained during the OLE through 60 weeks of ravulizumab treatment (Fig. 3). There was a statistically significant improvement from RCP baseline to Week 60 in all efficacy endpoints (Table 2): the LS (95% CI) mean changes in total MG-ADL and QMG total scores were − 4.0 (− 4.8, − 3.1; p < 0.0001); and − 4.1 (− 5.4, − 2.9; p < 0.0001), respectively (Table 2). Changes from OLE baseline in all endpoints showed continued numerical improvement (Fig. 4; Table 2). At Week 60, compared with RCP baseline, 42 (76.4%) patients had experienced an improvement of ≥ 3 points on the MG-ADL scale and 25 (49.0%) had experienced an improvement of ≥ 5 points on the QMG.

**Table 2:** Least-squares mean change (95% CI) in efficacy endpoints during the OLE (OLE analysis set)

	Ravulizumab–ravulizumab^a^ (n = 78)			Placebo–ravulizumab^b^ (n = 83)		
	RCP baseline to Week 60 (OLE Week 34)	OLE baseline to Week 60 (OLE Week 34)		RCP baseline to Week 60 (OLE Week 34)	OLE baseline to OLE Week 2 or 4^c^	OLE baseline to Week 60 (OLE Week 34)
MG-ADL total score	− 4.0 (− 4.8, − 3.1) p < 0.0001	− 0.3 (− 0.9, 0.3) p = 0.3095		− 3.3 (− 4.3, − 2.2) p < 0.0001	− 1.7 (− 2.4, − 1.0) p < 0.0001	− 1.7 (− 2.7, − 0.8) p = 0.0007
QMG total score	− 4.1 (− 5.4, − 2.9) p < 0.0001	− 0.9 (− 1.9, 0.0) p = 0.0555		− 3.8 (− 5.1, − 2.4) p < 0.0001	− 2.1 (− 3.0, − 1.1) p < 0.0001	− 3.1 (− 4.2, − 1.9) p < 0.0001
MG-QOL15r total score	− 5.0 (− 6.9, − 3.1) p < 0.0001	− 0.8 (− 1.8, 0.3) p = 0.1562		− 5.4 (− 7.3, − 3.5) p < 0.0001	− 3.5 (− 5.1, − 1.9) p < 0.0001	− 3.1 (− 4.8, − 1.4) p = 0.0005
Neuro-QoL Fatigue total score	− 10.2 (− 15.1, − 5.3) p < 0.0001	− 1.5 (− 5.0, 1.9) p = 0.3831		− 14.0 (− 18.6, − 9.4) p < 0.0001	− 9.3 (− 13.7, − 5.0) p < 0.0001	− 8.0 (− 12.3, − 3.6) p = 0.0005

CI, confidence interval; MG-ADL, Myasthenia Gravis–Activities of Daily Living; MG-QOL15r, revised 15-item Myasthenia Gravis Quality of Life; Neuro-QoL, Neurological Quality of Life; OLE, open-label extension; QMG, Quantitative Myasthenia Gravis; RCP, randomized controlled period

^a^Patients treated with ravulizumab during both the RCP and OLE. ^b^Patients treated with placebo during the RCP and ravulizumab during the OLE. ^c^OLE Week 2 for MG-ADL and QMG scores, OLE Week 4 for MG-QOL15r and Neuro-QoL Fatigue scores

In the placebo–ravulizumab group, rapid improvements (within 2–4 weeks) were observed in all efficacy endpoints following ravulizumab initiation (Figs. 3 and 4). Statistically significant improvements from OLE baseline were seen at the first OLE assessment point (2 weeks after ravulizumab initiation for MG-ADL and QMG scores and 4 weeks after ravulizumab initiation for MG-QOL15r and Neuro-QoL Fatigue scores). Improvements were sustained through Week 60, with LS (95% CI) mean changes from OLE baseline of − 1.7 (− 2.7, − 0.8; p = 0.0007) and − 3.1 (− 4.2, − 1.9; p < 0.0001) in total MG-ADL and QMG total scores, respectively (Table 2). At Week 60 (34 weeks after ravulizumab initiation), compared with OLE baseline, 25 (43.1%) patients had experienced an improvement of ≥ 3 points on the MG-ADL scale and 16 (32.7%) had experienced an improvement of ≥ 5 points in QMG score. At patients’ last assessments, such improvements were achieved by 33 (39.8%) and 23 (29.9%) patients, respectively.

During the OLE period assessed in this interim analysis, which included 161 patients, four patients in the placebo–ravulizumab group and six in the ravulizumab–ravulizumab group discontinued corticosteroid use; 26 patients in the placebo–ravulizumab group and 19 in the ravulizumab–ravulizumab group reduced their corticosteroid use. Two of the patients in the placebo–ravulizumab group initiated corticosteroid treatment and three increased their corticosteroid use; in the ravulizumab–ravulizumab group, two patients initiated corticosteroid use and six increased their use.

### Clinical deterioration events

In the ravulizumab–ravulizumab group, eight (9%) patients experienced 10 clinical deterioration events during the RCP and eight (10%) patients experienced 10 events during the OLE (Table 3). In the placebo–ravulizumab group, 15 (17%) patients experienced 26 clinical deterioration events during the RCP while receiving placebo compared with four (5%) patients experiencing five events during the OLE when receiving ravulizumab (Table 3).

**Table 3:** Clinical deterioration events experienced during the study

Clinical deterioration events^a^	Ravulizumab–ravulizumab		Placebo–ravulizumab	
	RCP (receiving ravulizumab) (n = 86)	OLE (receiving ravulizumab) (n = 78)	RCP (receiving placebo) (n = 89)	OLE (receiving ravulizumab) (n = 83)
Total				
Patients, n (%)	8 (9)	8 (10)	15 (17)	4 (5)
Events, n	10	10	26	5
MG crisis^b^				
Patients, n (%)	0	2 (3)^e^	1 (1)^f^	0
Events, n	0	3	1	0
Significant symptomatic worsening^c^				
Patients, n (%)	1 (1)	0	5 (6)^g^	1 (1)^f^
Events, n	1	0	6	1
Rescue therapy for health in jeopardy^d^				
Patients, n (%)	7 (8)	6 (8)	12 (13)	3 (4)
Events, n	9	7	19	4

Data for the full analysis set are shown for the RCP; data for the OLE analysis set are shown for the OLE

MG, myasthenia gravis; OLE, open-label extension; RCP, randomized controlled period

^a^Per-protocol-defined criteria for clinical deterioration event; a clinical deterioration event may have met more than one criterion, and patients may have experienced more than one event

^b^Defined as weakness severe enough to necessitate intubation or to delay extubation following surgery

^c^Defined as worsening to a score of 3, or a 2-point worsening from baseline, on any of the individual MG-ADL items other than double vision or eyelid droop, which in the investigator’s assessment was associated with significant symptomatic worsening

^d^Defined as the administration of rescue therapy to a patient whose health, in the opinion of the investigator, would be in jeopardy if rescue therapy were not given

^e^Both patients required rescue therapy

^f^Required rescue therapy

^g^Three patients (one patient on two occasions) required rescue therapy

**Fig. 5:**
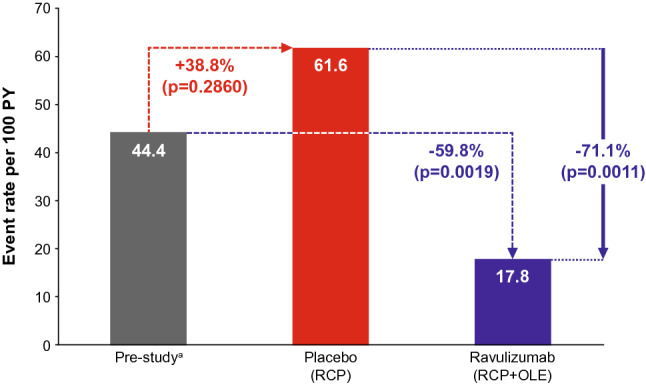
Clinical deterioration event rate per 100 patient-years. ^a^1-year period, events reported by investigators. The event rates were calculated using a generalized estimating equation Poisson regression repeated measures model with the number of events as the dependent variable, the logarithm of patient-years as the offset variable, and the study or phase indicator (pre-study, placebo, and ravulizumab RCP + OLE) as the factors, assuming a compound symmetry correlation structure. OLE, open-label extension; PY, patient-years; RCP, randomized controlled period

The clinical deterioration event rate per 100 patient-years was 44.4 in the 1-year period before the start of the study, 61.6 in patients receiving placebo during the RCP, and 17.8 in patients receiving ravulizumab in the RCP and OLE (Fig. 5). This corresponds to a reduction of 59.8% (p = 0.0019) for ravulizumab versus pre-study values and a reduction of 71.1% (p = 0.0011) for ravulizumab versus placebo (Fig. 5).

### Safety

Ravulizumab was generally well tolerated during both the RCP and OLE (Table 4). The most common adverse events were headache and diarrhea. Most adverse events (89%) were grades 1 or 2 in severity (Table 4). Event rates per 100 patient-years were higher in patients treated with ravulizumab than in those receiving placebo for abdominal pain, dizziness, and upper respiratory tract infection (Table 4). A total of 41 (24.3%) patients experienced a serious adverse event, of which six events (in five patients) were classified by the investigator as related to study treatment: one patient each with dysphagia, suppurative tendonitis, worsening MG, or erysipelas, and one patient with pneumonia and mitral valve stenosis. Four deaths occurred, all in patients receiving ravulizumab: three due to COVID-19 and one due to a spontaneous cerebral hemorrhage. All deaths were assessed by the investigators and confirmed by the sponsor to be unrelated to study treatment. No cases of meningococcal infection were reported. One case of meningitis with unknown etiology occurred at approximately 63 weeks after study entry (after the data cut-off date); cerebrospinal fluid (CSF) cultures for bacteria, *Mycobacterium tuberculosis*, and fungus, as well as CSF polymerase chain reaction analysis and blood cultures were all negative for bacterial meningitis. Serology results noted that *N. meningitidis* serogroup/serotype was not detected. The patient continued to participate in the study and to receive ravulizumab.

**Table 4:** Safety outcomes

AEs	Patients treated with ravulizumab during the RCP or OLE^a^		Patients treated with placebo during the RCP	
	n = 169, PY = 141.6		n = 89, PY = 43.0	
	Patients, n (%)	Events, n (rate per 100 PY)	Patients, n (%)	Events, n (rate per 100 PY)
Any AE	150 (88.8)	881 (622.0)	77 (86.5)	341 (793.1)
Related to trial agent^b^	58 (34.3)	146 (103.1)	30 (33.7)	61 (141.9)
Any AE, by severity^c^				
Grade 1	127 (75.1)	577^e^	66 (74.2)	250^e^
Grade 2	82 (48.5)	210^e^	30 (33.7)	70^e^
Grade 3	39 (23.1)	81^e^	14 (15.7)	20^e^
Grade 4	9 (5.3)	9^e^	1 (1.1)	1^e^
Grade 5	4 (2.4)	4^e^	0	0
Any SAE	41 (24.3)	75 (53.0)	14 (15.7)	16 (37.2)
Related to trial agent^b^	5 (3.0)	6^e^	4 (4.5)	4^e^
Death^d^	4 (2.4)	4^e^	0	0
AE reported in > 5% of patients in either group				
Headache	28 (16.6)	43 (30.4)	23 (25.8)	27 (62.8)
Diarrhea	23 (13.6)	26 (18.4)	11 (12.4)	15 (34.9)
Nausea	16 (9.5)	25 (17.7)	9 (10.1)	10 (23.3)
Fatigue	16 (9.5)	19 (13.4)	6 (6.7)	6 (14.0)
Back pain	16 (9.5)	16 (11.3)	5 (5.6)	5 (11.6)
Arthralgia	15 (8.9)	23 (16.2)	7 (7.9)	8 (18.6)
Nasopharyngitis	15 (8.9)	17 (12.0)	5 (5.6)	7 (16.3)
Urinary tract infection	15 (8.9)	17 (12.0)	4 (4.5)	5 (11.6)
Dizziness	14 (8.3)	19 (13.4)	3 (3.4)	3 (7.0)
COVID-19	9 (5.3)	13 (9.2)	3 (3.4)	4 (9.3)
Abdominal pain	9 (5.3)	11 (7.8)	0	0
Upper respiratory tract infection	9 (5.3)	10 (7.1)	2 (2.2)	2 (4.7)
Pyrexia	6 (3.6)	6 (4.2)	5 (5.6)	6 (14.0)
Infusion-related reaction	1 (0.6)	2 (1.4)	5 (5.6)	5 (11.6)

AE, adverse event; COVID-19, coronavirus disease 2019; OLE, open-label extension; PY, patient-years; RCP, randomized controlled period; SAE, serious adverse event

^a^Safety set: includes data available for all patients who received ≥ 1 dose of ravulizumab in the RCP or the OLE, up to Week 60 at data cut-off (9 November 2021)

^b^As determined by the investigator

^c^Graded according to National Cancer Institute Common Terminology Criteria for Adverse Events version 4.03

^d^Two deaths occurred during the RCP and two during the OLE (see text for details)

^e^Rate not calculated

## Discussion

The interim results of this long-term extension of the CHAMPION MG study demonstrate that the benefits of ravulizumab are sustained through 60 weeks in patients with anti-AChR antibody-positive gMG. In patients who received ravulizumab during the RCP, improvements in activities of daily living, muscle strength, fatigue, and quality of life were seen by Week 1 and sustained through 60 weeks of treatment.

There was a rapid and sustained beneficial response to ravulizumab in patients who switched from placebo to ravulizumab in the OLE, consistent with that observed for patients treated with ravulizumab during the RCP [26]. Statistically significant improvements from baseline were seen at the first assessment timepoints: 2 weeks after the start of therapy for the MG-ADL and QMG scores, and 4 weeks after the start of therapy for the MG-QOL15r and Neuro-QoL Fatigue scores. There were also statistically significant improvements in each scale both from RCP baseline to Week 60 and from OLE baseline to Week 60 (34 weeks of ravulizumab treatment for the placebo–ravulizumab group), the latter of which was achieved despite the high placebo response observed in the primary analysis of the RCP [26].

The criteria for response in the CHAMPION MG study were an improvement of ≥ 3 points on the MG-ADL scale or ≥ 5 points in the QMG score; both exceed the minimal clinically important difference generally accepted for these measures (i.e. ≥ 2 points on the MG-ADL scale and ≥ 3 points in the QMG score) [32, 33]. In the ravulizumab–ravulizumab group, 76.4% of patients were considered responders according to the study MG-ADL criterion and 49.0% according to the study QMG criterion at Week 60 compared with RCP baseline. In comparison, at the 26-week assessment, the response rate was 56.7% for MG-ADL and 30.0% for QMG [26]. These trends suggest that the response rate may increase with prolonged treatment, as seen in the REGAIN trial OLE with eculizumab [20, 34, 35]. In the placebo–ravulizumab group, 43.1% and 32.7% of patients were considered to be MG-ADL and QMG responders, respectively, at Week 60 compared with OLE baseline, after up to 34 weeks of treatment. These response rates are similar to those observed for the ravulizumab–ravulizumab group after 26 weeks, albeit slightly lower for MG-ADL. It is not clear why some patients did not respond within the timeframe examined in this analysis. As mentioned above, the response criteria applied in the study were stringent and exceeded the accepted minimal clinically important difference for MG-ADL and QMG scores [32, 33]. At present, no clinical indicators or markers are established for response to complement inhibitor therapy in MG and further research is required in this area.

Changes to immunosuppressant use were permitted only during the OLE. During the OLE period assessed in the interim analysis (up to 34 weeks of ravulizumab treatment) several patients were able to decrease or discontinue their corticosteroid use, suggesting that adults with gMG who are treated with ravulizumab may be able to reduce their regular corticosteroid dosage, and hence lessen the adverse-event burden associated with such treatment.

During the RCP, the proportion of patients experiencing a clinical deterioration event was approximately halved in the ravulizumab group compared with the placebo group [26]. After patients who received placebo in the RCP were switched to ravulizumab in the OLE, the number of clinical deterioration events and the proportion of patients experiencing these events decreased. Moreover, statistically significant reductions in exposure-adjusted clinical deterioration event rates were observed in ravulizumab-treated patients across the RCP and OLE periods compared with both pre-study rates and rates in patients while receiving placebo. Given the short- and long-term health and social impacts of clinical deterioration events on people with gMG, these results demonstrate an important potential benefit for patients treated with ravulizumab.

The results of this interim analysis support the efficacy of the weight-based dosing regimen with an 8-week dosing interval. This regimen is used for other approved indications and has been shown to provide immediate, sustained, and complete C5 inhibition over the whole dosing interval [36]. Pharmacokinetic and pharmacodynamic analyses have also demonstrated immediate (by end of first infusion), complete, and sustained inhibition of terminal complement in patients in the CHAMPION MG study [26]. A stable and sustained therapeutic effect is essential for a chronic and fluctuating disease such as gMG.

The safety profile of ravulizumab in the long-term extension period was consistent with that in the 26-week RCP [26]. Abdominal pain, dizziness, and upper respiratory tract infection were the only adverse events reported to occur at a higher rate in patients treated with ravulizumab in the RCP or OLE than in those receiving placebo in the RCP. Of the four deaths that occurred in patients receiving ravulizumab, three were related to COVID-19 infection; there was no record that any of the three patients had received COVID-19 vaccination. There is no evidence that ravulizumab treatment increases the risk of mortality in patients with COVID-19 infection. In a randomized, open-label study investigating ravulizumab plus best supportive care versus best supportive care alone in patients with severe COVID-19, no difference in overall survival was observed between the two groups [37]. The fourth death that occurred during the CHAMPION MG study was due to cerebral hemorrhage and occurred in a patient with a medical history of atrial fibrillation, diabetes mellitus, hyperlipidemia, and hypertension, who was receiving therapy with an oral anticoagulant. None of these deaths was considered to be related to ravulizumab treatment (for more information see Supplementary Table S1). The safety profile is also consistent with studies of ravulizumab in PNH and aHUS [22, 23], and with the safety profile of eculizumab in gMG and other disorders such as PNH, aHUS, and neuromyelitis optica spectrum disorder [19, 20, 38–41]. No new safety signals were identified in the CHAMPION MG study and no cases of meningococcal infection were reported.

The findings of the CHAMPION MG study of ravulizumab are consistent with those of the REGAIN study of eculizumab in anti-AChR antibody-positive gMG [19, 20]. In that study, rapid and statistically significant improvements versus placebo in outcomes including activities of daily living, muscle strength, and functional ability were seen during the randomized, placebo-controlled period of the study [19]. These efficacy findings were sustained for up to 3 years during the open-label extension period [20]. The results of both CHAMPION MG and REGAIN provide evidence that inhibiting complement protein C5 is an efficacious and rational therapeutic target in patients with anti-AChR antibody-positive gMG. Ravulizumab provides the additional benefit that therapeutic serum concentrations are maintained over an 8-week dosing interval [21], potentially improving convenience by reducing the number of annual infusions from 26 with eculizumab to 6–7 with ravulizumab.

Strengths of the CHAMPION MG study have been previously described [26]. A wide variety of patients were enrolled, with broad ranges of disease severity, standard treatments received before the trial, disease duration, and geographic background. Also, in contrast to REGAIN, patients enrolled in CHAMPION MG were not required to meet criteria defining them as being refractory to previous MG treatments. The relevance of the outcome measures used, including both patient- and physician-reported assessments, was also a strength. The main limitation of the long-term extension is its open-label design, which might contribute to an over-estimation of effectiveness. However, the dosing schedule at the start of the OLE was designed to preserve study blinding when the initial response to ravulizumab was being measured in patients who had previously received placebo. Selection bias is a potential limitation of extension studies, whereby only patients who respond well to therapy during the main study go forward to the extension phase. However, 161 patients out of 162 who completed the RCP elected to enter the OLE, so it is unlikely that this type of selection bias substantially influenced the results.

In conclusion, the interim analysis demonstrates that patients who initiated ravulizumab at RCP baseline sustained their improvements for up to 60 weeks, while patients who switched from placebo to ravulizumab at OLE baseline achieved a rapid and sustained improvement in their MG symptoms, consistent with that observed in patients who initiated ravulizumab treatment at the start of the study. The study also demonstrates that ravulizumab has the potential to decrease the rate of clinical deteriorations. The safety profile was consistent with the known safety profile of ravulizumab and no new safety signals were identified. Overall, these findings support the sustained clinical effectiveness and long-term safety of ravulizumab, administered every 8 weeks, in adults with anti-AChR antibody-positive gMG.

## Supplementary Information

Below is the link to the electronic supplementary material.

Supplementary file1 (PDF 236 KB)

## Data Availability

Alexion, AstraZeneca Rare Disease will consider requests for disclosure of clinical study participant-level data provided that participant privacy is assured through methods like data de-identification, pseudonymization, or anonymization (as required by applicable law), and if such disclosure was included in the relevant study informed consent form or similar documentation. Qualified academic investigators may request participant-level clinical data and supporting documents (statistical analysis plan and protocol) pertaining to Alexion-sponsored studies. Further details regarding data availability and instructions for requesting information are available in the Alexion Clinical Trials Disclosure and Transparency Policy at https://alexionclinicaltrials.com/Disclosure-and-Transparency-Policy. Link to Data Request Form: https://alexion.com/contact-alexion/medical-information.
