# Supplementary material for: Long-term efficacy and safety of ravulizumab in adults with anti-acetylcholine receptor antibody-positive generalized myasthenia gravis: results from the phase 3 CHAMPION MG open-label extension
Source: J Neurol. 2023 Apr 27;270(8):3862–75. doi: 10.1007/s00415-023-11699-x (PMC10134722; doi:10.1007/s00415-023-11699-x)
Supplement: Supplementary file 1 — Supplementary file1 (PDF 236 KB) [file 415_2023_11699_MOESM1_ESM.pdf]

Supplementary information

Long-term efficacy and safety of ravulizumab in adults with anti-acetylcholine receptor antibody-positive generalized myasthenia gravis: results from the phase 3 CHAMPION MG open-label extension

Journal of Neurology

Andreas Meisel,[1] Djillali Annane,[2] Tuan Vu,[3] Renato Mantegazza,[4] Masahisa Katsuno,[5] Rasha Aguzzi,[6] Glen Frick,[6] Laura Gault,[6] James F Howard Jr,[7] and the CHAMPION MG Study Group

[1]Charité Universitätsmedizin Berlin, Berlin, Germany; [2]Hôpital Raymond Poincare, Garches, France; [3]University of South Florida Morsani College of Medicine, Tampa, FL, USA; [4]Fondazione IRCCS Istituto Neurologico Carlo Besta, Milan, Italy; [5]Nagoya University Graduate School of Medicine, Nagoya, Japan; [6]Alexion, AstraZeneca Rare Disease, Boston, MA, USA; [7]The University of North Carolina, Chapel Hill, NC, USA.

Corresponding author

Andreas Meisel; Email: andreas.meisel@charite.de

Contents

List of study investigators 3

Table S1. Summary of deaths in the CHAMPION MG study 15

List of study investigators

Austria:

Kepler University Clinic, Linz: Principal investigator: Michael Guger; Sub-investigators: Rainer Dormann, Stephan Eger, Lukas Kellermair, Eva Lenzenweger; Study coordinator: Sandra Rathmaier

Medical University of Vienna AKH, Vienna: Principal investigator: Friedrich Zimprich; Sub-investigators: Hakan Cetin, Jakob Rath, Matthias Tomschik, Rosa Weng, Gudrun Zulehner; Study coordinator: Sonja Wieszmuellner

Canada:

London Health Sciences Centre, London, ON: Principal investigator: Michael W. Nicolle; Sub-investigator: Anita Florendo-Cumbermack; Study coordinator: Denise Hulley

Montreal Neurological Institute and Hospital, Montreal, QC: Principal investigator: Angela Genge; Sub-investigators: Maxime Berube, Rami Massie; Study coordinators: Xin Dong, Raquel Farias, Dylan Sembinelli

Toronto General Hospital University Health Network, Toronto, ON: Principal investigator: Vera Bril; Sub-investigator: Hans Katzberg; Main study coordinator: Eduardo Ng; Back-up study coordinators: Lubna Daniyal, Shabber Mannan, Raghu Raman, Ritesh Rohan, Evelyn Sarpong

University of Alberta Hospital, Edmonton, Alberta: Principal investigator: Zaeem A. Siddiqi; Sub-investigators: Mohammed Wassif Hussain, Cecile Phan; Study coordinators: Muhammed Abid Alvi, Hussain Faraz

University of Calgary, Calgary, Alberta: Principal investigator: Lawrence Korngut; Sub-investigators: Rodney Li Pi Shan, Theodore Mobach; Study coordinators: Jose Martinez, Janet Petrillo

Czech Republic:

Faculty Hospital Ostrava-Poruba, Ostrava: Principal investigator: Jana Junkerova; Sub-investigators: Eva Kovalova, Martin Sabela; Study coordinators: Michaela Sobkova, Miluse Roznovska

General University Hospital, Prague: Principal investigator: Michaela Tyblova; Sub-investigators: Michala Jakubikova, Irena Rysankova, Iveta Novakova; Study coordinators: Ivana Jurajdova, Hana Kulhava

Thomayerova Nemocnice, Prague: Principal investigator: Petr Ridzon; Sub-investigator: Katerina Matejova; Study coordinators: Katerina Slovakova, Jana Bezdecna, Marketa Dusart, Jiri Skopek

University Hospital Brno, Brno: Principal investigator: Stanislav Vohanka; Sub-investigators: Magda Horakova, Tomas Horak; Study coordinator: Katerina Havelkova

Denmark:

Aalborg University Hospital, Aalborg: Principal investigator: Izabella Obál; Sub-investigator: Lotte Vinge; Study coordinator: Anita Palsgard Klyver

Aarhus University Hospital, Aarhus: Principal investigator: Henning Andersen; Sub-investigators: Lotte Levison, Jan Lykke Scheel Thomsen; Study coordinators: Tina Maria Bonde Christiansen, Dorthe Sønderborg Welch

Rigshospitalet, Copenhagen: Principal investigator: John Vissing; Sub-investigators: Joan Lilja Sunnelyg Hoejgaard, Nanna Witting, Sonja Holm Yildiz, Nicolai Preisler: Study coordinators: Zillah Joensen

France:

CHU de Lille - Hopital Boulevard Roger Salengro, Lille: Principal investigator: Celine Tard; Sub-investigator: Jean Baptiste Davion; Study coordinators: Peggy Lecointe, Valerie Santraine

CHU la Timone, Marseille: Principal coordinator: Shahram Attarian; Sub-investigators: Emmanuelle Salort-Campana, Emilien Delmont, Aude-Marie Grapperon, Ludivine Kouton; Study coordinator: Saran Diallo; Back-up study coordinator: Nacime Heddadji

CHU Nice - Hopital Pasteur 2, Nice: Principal investigator: Saskia Bresch; Sub-investigators: Mikael Cohen, Christine Lebrun-Frenay; Study coordinator: Ruxanda Bodisteanu

Hospital Raymond Poincaré, Garches: Principal investigator: Djillali Annane; Sub-investigators: Suzanne Amthor, Bernard Clair, Diane Friedman, David Olikowsky; Study coordinator: Maimouna Kane

Germany:

Charité Universitätsmedizin Berlin, Berlin: Principal investigator: Andreas Meisel; Sub-investigators: Lea Gerischer, Sarah Hoffman, Benjamin Hotter, Sophie Lehnerer, Anne Lesemann, Frauke Stascheit; Study coordinators: Claudia Heibutzki, Dike Remstedt

Klinikum Würzburg Mitte, Würzburg: Principal investigator: Mathias Maeurer; Sub-investigators: Marie Gruendahl, Christoph Uibel; Study coordinator: Ekaterina Titova

Medizinische Hochschule Hannover, Hannover: Principal investigator: Martin Stangel; Sub-investigators: Thomas Skripuletz, Dominica Ratuszny, Philipp Schwenkenbecher; Study coordinators: Sikinika Hache, Karin Fricke

Universitätsklinikum Halle, Halle: Principal investigator: Alexander Emmer; Sub-investigator: Alexander Mensch; Study coordinator: Andrea Lehman

Universitatsklinikum Hamburg-Eppendorf, Hamburg: Principal investigator: Amir Golsari; Sub-investigators: Milani Deb-Chatterji, Mathias Gelderblom; Study coordinators: Hannes Appelbohm, Christoph Brosinski, Kirsten Jaramillo, Dagmar Otto

University Hospital Essen, Essen: Principal investigator: Tim Hagenacker; Sub-investigators: Saskia Bolz, Kathrin Kizina, Christine Stadtler, Benjamin Stolte, Andreas Thimm, Andreas Totzeck; Study coordinator: Jaqueline Lipka

University Hospital Münster, Department of Neurology, Münster: Principal investigator: Heinz Wiendl; Sub-investigators: Nora Buenger, Lucienne Kirstein, Luisa Klotz, Jan Luenemann, Marc Pawlitzki, Tobias Ruck; Study coordinators: Karla Musiolik, Anna Lammerskitten, Carolin Risau, Claudia Schwering

University of Leipzig Medical Center, Leipzig: Principal investigator: Wolfgang Koehler; Sub-investigators: Petra Baum, Hannes Roicke, Astrid Unterlauft, Caroline Bergner; Study coordinators: Eike Haensel, Andrea Kalb, Bianca Meilick, Mandy Reuschel, Heidi Schilling

Israel:

Rambam Health Care Campus, Haifa: Principal investigator: Yitzhak Schiller; Previous principal investigator: David Yarnitsky; Sub-investigators: Tahani Alsheikh, Sameh Ayoub, Chen Buxbaum, Mohamed Khateb; Study coordinators: Svetlana Afanasiev, Meital Tesler

The Tel Aviv Sourasky Medical Center, Tel Aviv: Principal investigator: Arnon Karni; Sub-investigators: Keren Regev, Ifat Vigiser; Study coordinators: Avigail Hindi, Irina Komarov

Italy:

A.O.U. Policlinico "Gaetano Martino", Messina: Principal investigator: Carmelo Rodolico; Sub-investigators: Carmen Bonanno, Guilia Nicocia, Alessia Pugliese; Study coordinators: Alessia Pugliese, Antonino Lupica, Carmen Bonanno, Giulia Nicocia

ASST Papa Giovanni XXIII, Bergamo: Principal investigator: Manlio Sgarzi; Sub-investigators: Giorgia Camera, Emanuela Agazzi; Study coordinator: Giorgia Camera, Bianca Taddei

IRCCS AOU San Martino, Genova: Principal investigator: Angelo Schenone; Sub-investigators: Marina Grandis, Sara Massucco, Angela Zuppa; Study coordinator: Marta Traversa

IRCCS Fondazione Policlinico Universitario Agostino Gemelli, Rome: Principal investigator: Amelia Evoli; Sub-investigators: Raffaele Iorio, Gianvito Masi, Gabriele Monte, Gregorio Spagni; Study coordinators: Raffaele Iorio, Gianvito Masi, Gregorio Spagni

IRCCS Istituto delle Scienze Neurologiche dell'Azienda USL di Bologna, Bologna: Principal investigator: Rocco Liguori; Sub-investigators: Maria Pia Giannoccaro, Veria Vacchiano; Study coordinators: Maria Pia Giannoccaro, Silvia De Luca, Claudia Boninsegna

IRCCS Istituto Neurologico Carlo Besta, Milan: Principal investigator: Renato Mantegazza; Sub-investigators: Carlo Antozzi, Silvia Bonanno, Rita Frangiamore, Riccardo Giossi, Lorenzo Maggi, Fiammetta Vanoli; Study coordinator: Elena Rinaldi

Sapienza University of Rome, NESMOS Department, Sant'Andrea Hospital, Rome: Principal investigator: Giovanni Antonini; Sub-investigators: Girolamo Alfieri, Laura Fionda, Matteo Garibaldi, Antonio Lauletta, Luca Leonardi, Stefania Morino, Fiammetta Vanoli; Study coordinators: Laura Fionda, Matteo Garibaldi, Luca Leonardi, Stefania Morino

Japan:

Chiba University Hospital, Chiba City, Chiba: Principal investigator: Akiyuki Uzawa; Sub-investigators: Yukiko Ozawa, Manato Yasuda; Study coordinator: Youko Kaneko

Hanamaki General Hospital, Hanamaki, Iwate: Principal investigator: Kimiaki Utsugisawa; Sub-investigator: Yuriko Nagane; Study coordinators: Ayumi Kameda, Yuka Sato, Tomoko Tsuda, Yumiko Yokota

Izumi City General Hospital, Izumi, Osaka: Principal investigator: Yukihiro Hamada; Sub-investigators: Shuichi Ueno, Masaki Yamana; Study coordinator: Mitsuki Hasuda

Juntendo University Hospital, Tokyo: Principal investigator: Kazumasa Yokoyama; Sub-investigators: Taku Hatano, Yutaka Oji, Yuji Tomizawa; Study coordinator: Shiratori Atsuko

Kanazawa University Hospital, Kanazawa, Ishikawa: Principal investigator: Yutaka Furukawa; Previous principal investigator: Kazuo Iwasa; Sub-investigators: Kazuo Iwasa, Daiki Muramatsu, Yasutake Tada

Kansai Medical University Medical Center, Moriguchi, Osaka: Principal investigator: Takayuki Kondo; Sub-investigators: Yuya Shinoto, Kumi Itani; Study coordinators: Satoko Kuroda, Azusa Kawaguchi

Keio University Hospital, Tokyo: Principal investigator: Shigeaki Suzuki; Sub-investigator: Kei Ishizuchi, Kenji Kufukihara, Kensuke Okada, Munenori Oyama; Study coordinators: Tomoka Miyazaki

Kindai University Hospital, Osakasayama, Osaka: Principal investigator: Makoto Samukawa; Sub-investigators: Yuta Fukumoto, Rino Inada, Miyuki Morikawa, Yuko Yamagishi, Keisuke Yoshikawa; Study coordinator: Yoshimi Ogaki

Kyushu University Hospital, Fukuoka City, Fukuoka: Principal investigator: Takuya Matsushita; Sub-investigators: Noriko Isobe, Dai Matsuse, Hidenori Ogata, Mitsuru Watanabe; Study coordinator: Yukiko Yoshizuru

Nagasaki University Hospital, Nagasaki City, Nagasaki: Principal investigator: Akira Tsujino; Sub-investigators: Teiichiro Miyazaki, Atsushi Nagaoka, Tomoaki Shima, Hirokazu Shiraishi, Shunsuke Yoshimura; Study coordinators: Yuri Fukushige, Kazumi Takada

Nagoya University Hospital, Nagoya, Aichi: Principal investigator: Masahisa Katsuno; Sub-investigators: Atsushi Hashizume, Daisuke Ito, Yoshiyuki Kishimoto, Shinichiro Yamada; Study coordinators: Chiharu Ikeda, Yuko Noda, Megumi Sasaki

National Hospital Organization Hokkaido Medical Center, Sapporo, Hokkaido: Principal investigator: Naoya Minami; Sub-investigators: Itaru Amino, Sachiko Akimoto, Masaaki Niino, Yusei Miyazaki, Fumihito Nakano; Study coordinators: Kazuya Takatsu, Reika Umetsu

National Hospital Organization Osaka Toneyama Medical Center, Toyonaka, Osaka: Principal investigator: Keiko Toyooka; Sub-investigators: Yakuyuki Endo, Yuto Hayashi, Hiroko Kimura, Kimiko Inoue, Misa Matsui, Chiaki Mori, Daisuke Nakatsu, Hiroto Namba, Tomoko Saito, Toshio Saito, Motohiro Sudo, Natsuki Tanaka, Tomohiro Yata

Niigata University Medical & Dental Hospital, Niigata City, Niigata: Principal investigator: Izumi Kawachi; Sub-investigators: Akihiro Nakajima, Etsuji Saji, Takayoshi Tokutake,

Takahiro Wakasugi, Kaori Yanagawa; Study coordinators: Satomi Ikarashi, Moemi Minagawa, Ayumi Namekata, Junko Sato

Osaka University Hospital, Suita, Osaka: Principal investigator: Hideki Mochizuki; Sub-investigators: Tomoya Kubota, Masayuki Nakamori, Tatsusada Okuno, Masanori Takahashi; Study coordinators: Ai Kawakami, Namie Taichi, Hiromi Tanaka

Saitama Medical Center, Kawagoe, Saitama: Principal investigator: Kenichi Kaida; Previous principal investigator: Kyoichi Nomura; Sub-investigators: Wataru Hara, Keita Ishizuka, Shoko Izaki, Shinya Narukawa, Satoru Oji, Kouhei Sugimoto, Masato Suzuki, Satoru Tanaka, Norihito Yoshida; Study coordinators: Momoko Kaneko, Yoshiko Murakami, Masakazu Tsukagoshi

Sapporo Medical University Hospital, Sapporo, Hokkaido: Principal investigator: Daisuke Yamamoto; Previous principal investigator: Tomihiro Imai; Sub-investigators: Kazuna Ikeda, Tomohiro Imai; Study coordinators: Rie Koshika, Honoka Yamaguchi

Sendai Medical Center, Sendai, Miyagi: Principal investigator: Yasushi Suzuki; Sub-investigators: Sousuke Harigae, Kimiko Inoue, Genya Watanabe; Study coordinators: Sayaka Ishida, Mitsuo Yata

The University of Tokyo Hospital, Tokyo: Principal investigator: Tatsushi Toda; Sub-investigators: Hiroyuki Ishiura, Reiko Kawasaki, Ayako Koguchi, Akatsuki Kubota, Kaori Sakuishi; Study coordinator: Mami Okanaga

Tokyo Medical and Dental University, Medical Hospital, Tokyo: Principal investigator: Yoichiro Nishida; Sub-investigators: Takaaki Hattori, Satoru Ishibashi, Taro Ishiguro, Takuya Ohkubo, Nobuo Sanjo, Yohsuke Yagi, Takanori Yokota; Study coordinators: Yuuki Okubo, Mayuko Hogari

Yamaguchi University Hospital, Ube, Yamaguchi: Principal investigator: Takashi Kanda, Sub-investigators: Susumu Fujihara, Yuichi Fujimoto, Miwako Fujisawa, Masaya Honda, So Kanda, Motoharu Kawai, Michiaki Koga, Toshiko Maeda, Kinya Matsuo, Jo Nemoto, Mariko Oishi, Masatoshi Omoto, Namiko Oshibe, Hironori Sano, Yasuteru Sano, Ryota Sato, Fumitaka Shimizu, Shiori Takahashi, Yukio Takeshita, Nanami Yamanaka; Study coordinator: Natsumi Unezaki

Netherlands:

Amsterdam UMC, Location AMC, Amsterdam: Principal investigator: Anneke J. Van der Kooi; Sub-investigator: Filip Eftimov, Angela Langerak; Study coordinator: Tamar Gibson

Leiden University Medical Center (LUMC), Leiden: Principal investigator: Jan J.G.M. Verschuuren; Sub-investigators: Kevin R. Keene, Annabel M. Ruiter, Martijn R. Tannemaat; Study coordinators: Marjolein J. van Heur-Neuman, Anne-Marie M.G.H. Peters

Portugal:

Centro Hospitalar Universitario do Porto, Hospital Santo Antonio, Porto: Principal investigator: Ernestina Santos; Sub-investigators: Ana Martins da Silva, Raquel Samoes, Ana Paula Sousa; Study coordinator: Daniela Boleixa

Republic of Korea:

Asan Medical Center, Seoul: Principal investigator: Young-Min Lim; Sub-investigators: Eun Jae Lee, Hye Weon Kim, Kyu Yoon Chung; Study coordinators: Eun Ji Kang, Ae Ran Song

Konkuk University Medical Center, Seoul: Principal investigator: Jeeyoung Oh; Sub-investigators: Kyomin Choi, Hee Jin Kim, Dayoung Kim; Study coordinator: Hyunjoo Jeong

Korea University Anam Hospital, Seoul: Principal investigator: Byung-Jo Kim; Sub-investigators: Seol-Hee Baek, Hayom Kim, Joo Hye Sung, Jinwoo Park, Jeong Hwa Rho; Study coordinators: Jin Hee Hwang, Se Young Jeong

Kyungpook National University Chilgok Hospital; Daegu: Principal investigator: Jin-Sung Park; Sub-investigators: Jaechun Hwang; Study coordinators: Yu Jeong Noh, Jia Kim, Gibok Kwon

Pusan National University Yangsan Hospital, Yangsan: Principal investigators: Jin-Hong Shin; Sub-investigators: Minsung Kang, Eun Hye Oh; Study coordinators: So-young Jang, Jiyung Jeong

Samsung Medical Center, Seoul: Principal investigator: Byoung Joon Kim; Sub-investigators: Woo Kyo Jeong, Ju-Hong Min, Jiah Kim, Hye Mi Kwon, Hye Jung Lee, Jaehong Park, Jong Hwa Shin; Study coordinator: Hyesun Kang

Seoul National University Hospital, Seoul: Principal investigator: Sung-Min Kim; Sub-investigators: Je-Young Shin; Jung-Joon Sung; Study Coordinator: Dakyung Wei

Severance Hospital, Yonsei University Health System, Seoul: Principal investigator: Ha Young Shin; Previous principal investigators: Seung Woo Kim, Seung Min Kim; Sub-investigators: Jinhyuk Cho, Hye Yoon Chung, In Gun Hwang, Kihooh Kim, Sohyeon Kim, Sooyoung Kim, Jee Eun Lee, Hyun Ji Lyou, Seung Woo Kim; Study coordinator: Yaein Kim

Spain:

Hospital Clinic de Barcelona, Barcelona: Principal investigator: Albert Saiz; Sub-investigators: Yolanda Blanco, Sara Llufriu, Eugenia Martinez-Hernandez, Carmen Montejo Gonzalez, Maria Sepulveda; Study coordinator: Montse Artola

Hospital Clinico San Carlos, Madrid: Principal investigator: Antonio Guerrero Sola; Sub-investigators: Lucia Galan Davila, Alejandro Horga Hernandez, Lorenzo Silva Hernandez, Vanesa Veronica Pytel; Study coordinator: Marta Palacios

Hospital Clinico Universitario Virgen de la Arrixaca, Murcia: Principal investigator: Jose Eustasio Meca Lallana; Sub-investigators: Ester Carreon Guarnizo, Gabriel Valero Lopez; Study coordinators: Francisca Iniesta, Encarnacion Aznar

Hospital Sant Pau i la Santa Creu, Barcelona: Principal investigator: Isabel Illa Sendra; Sub-investigators: Rodrigo Alvarez Velasco, Elena Cortes Vicente, Luis Antonio Querol Gutierrez; Study coordinators: Luis Antonio Querol Gutierrez, Clara Tejada, Rodrigo Alvarez Velasco, Nuria Vidal

Hospital Universitari Vall d'Hebron, Barcelona: Principal investigator: Maria Salvadò Figueras; Previous principal investigator: Joseph Gamez Carbonell; Sub-investigators:

Alejandro Martinez De La Ossa Vela; Raul Juntas Morales Alejandro, Alba Sierra Marcos, Antonio Palasi, Daniel Sanchez-Tejerinas; Study coordinators: Carla Aguilar, Anna Canovas, Gisela Gili, Estefania Soler Mendo, Sara Quiñoa

Hospital Universitario Cruces, Vizcaya: Principal investigator: Jose Eulalio Barcena Llona; Sub-investigators: Sabas Boyero Duran, Amaia Gonzalez Eizaguirre, Amaia Jauregui Burrutia; Study coordinator: Atsegine Canga Garces

Hospital Universitario de Bellvitge, Barcelona: Principal investigator: Carlos Casasnovas Pons; Sub-investigators: Moises Morales de la Prida, Velina Nedkova Hristova, Valentina Velez Santamaria; Study coordinator: Elena Fabra

Hospital Universitario Ramon y Cajal, Madrid: Principal investigator: Jaime Masjuan Vallejo; Sub-investigators: Nuria Garcia Barragan, Inigo Corral Corral, Juan Carlos Martinez Castrillo; Study coordinator: Otilia Navarro

Hospital Universitario y Politecnico La Fe de Valencia, Valencia: Principal investigator: Luis Bataller Alberola; Sub-investigators: Nuria Muelas Gomez, Teresa M. Sevilla Mantecon; Study coordinator: Paula Lizandra

La Paz University Hospital, Madrid: Principal investigator: Exuperio Diez Tejedor; Sub-investigators: Mireya Fernandez-Fournier Fernandez, Javier Amos Membrilla Lopez, Maria Sastre Real, Francisco Javier Rodriguez de Rivera Garrido; Study coordinator: Beatriz Chamoro

Switzerland:

University Hospital Zürich, Zürich: Principal investigator: Konrad P. Weber; Sub-investigators: Fabienne C. Fierz, Marianne Schesny, Bettina Schreiner, Yulia Valko; Study coordinators: Tanja Schmueckle Meier, Monica Spitaleri, Diana Verner-Ruckstuhl

United States of America:

Advanced Neurosciences Research, LLC, Fort Collins, CO: Principal investigator: Augusto A. Miravalle; Sub-investigators: Tamara A. Miller, Jerry D. Nash, Jill M. Olson; Study coordinators: Stephanie De Angelis (Kallsen), Jennifer Gentilcore, Kelley Sage

Ascension St. Francis Hospital Center for Neurological Diseases, Milwaukee, WI: Principal investigator: Bhupendra O. Khatri; Sub-investigators: Anna Baker, Kaylan Fenton, Virendra Misra, Lisa Sershon; Study coordinators: Tayo Olapo, Emily Lindman

Augusta University, Augusta, GA: Principal investigator: Michael H. Rivner; Sub-investigators: Benjamin Barnes, Kristy Bouchard, Diane Manghram, Brandy Quarles; Study coordinators: Kristy Bouchard, Brandy Quarles

Austin Neuromuscular Center, Austin, TX: Principal investigator: Yessar M. Hussain; Sub-investigator: Mariana Varga; Study coordinators: Emil Hussain, Nawar Hussin

Baptist Health Lexington, Lexington, KY: Principal investigator: James Winkley; Sub-investigators: Gregory Cooper, Alyssa Winebrenner; Study coordinators: Julie Ayers, Melissa Barnes, Jennifer Buntain, Emily Keefe, Taylor Lashbrook

Cedars-Sinai Medical Center, Los Angeles, CA: Principal investigator: Richard Lewis; Sub-investigators: Robert Baloh, Matthew Burford, Jillian Doherty, Carolyn Prina, Viviana Valencia; Study coordinators: Jillian Doherty (primary), Carolyn Prina, Nickie Toloueenia, Viviana Valencia, Koral Wheeler

Central Texas Neurology Consultants, Round Rock, TX: Principal investigator: Adam D. Horvit; Sub-investigators: Edward Fox, Lori Mayer; Study coordinator: Koni Lopez

Cleveland Clinic, Cleveland, OH: Principal investigator: Yuebing Li; Sub-investigator: Robert Marquardt; Study coordinators: Irys Caristo, Debbie Hastings

Clinical Trials of South Carolina, Charleston, SC: Principal investigator: David E. Stickler; Sub-investigators: Lauren Barsan, Patricia Myers, Rachael Zealy; Study coordinators: Deanna Lambert, Nathelia O'Banner

Colorado Springs Neurological Associates; Colorado Springs, CO: Principal investigator: Kimberly Wagner; Previous principal investigator: Kevin Scott; Sub-investigators: Christen Kutz, Kristen Moreno; Study coordinators: Aspen Beck, Jodi Ventimiglia

Duke University Medical Center, Durham, NC: Principal investigator: Vern C. Juel; Sub-investigator: Lisa Hobson-Webb; Study coordinator: Kate Beck

Forbes Norris MDA/ALS Research Center, San Francisco, CA: Principal investigator: Jonathan S. Katz; Sub-investigators: Liberty Jenkins, Robert Miller; Study coordinators: Marguerite Engel, Jennifer Milan, Ahalya Prakash

Georgetown University Hospital, Washington, DC: Principal investigator: Shakti Nayar; Previous principal investigator: Joseph M. Choi; Study coordinators: Lexy Ahmad, Sarai Bartlett, Zsofia Parragh

HonorHealth Neurology/Phoenix Neurological Associates Ltd., Scottsdale, AZ: Principal investigator: Todd Levine; Sub-investigators: Anne Hatch, Harry Tamm; Study coordinators: Camille Fajardo, Tori Haggett, Jennifer Pruitt, Mackenzie Steinbach

Hospital for Special Surgery, New York, NY: Principal investigator: Dale Lange; Sub-investigators: Samantha Bock, Brittaney Codelia, Shara Holzberg, Misha Hooda, Elizabeth Ng, Pantelis Pavlakis, Jeffrey Schachter, Mona Shahbazi

Houston Methodist Hospital, Houston, TX: Principal investigator: Ericka P. Greene; Sub-investigators: Ashley Anderson, Stanley Appel, Patricia Mendoza, Sheetal Shroff, R. Glenn Smith, Jason Thonhoff, Niloofar Yari; Study coordinator: Patricia Mendoza

Indiana University, Indianapolis, IN: Principal investigator: Cynthia Bodkin; Sub-investigators: Adam Comer, John Emmett, John Kincaid, Robert Pascuzzi; Study coordinators: Lara Ann Boulton, Sandra Guingrich, Angela Micheels, Amy Perkins

Johns Hopkins Outpatient Center, Baltimore, MD: Principal investigator: Vinay Chaudhry; Sub-investigator: Mohammad Khoshnoodi; Study coordinator: Betsy Mosmiller

Lahey Hospital and Medical Center, Burlington, MA: Principal investigator: Doreen Ho; Sub-investigators: Erin Clark, Lee-Anne Lipert, Michal Vytopil; Study coordinators: Dayana Blanchet (primary), Erin Clark, Tanya Fennell

Las Vegas Clinic, Las Vegas, NV: Principal investigator: Jonathan H. McKinnon; Sub-investigator: Naya McKinnon, Study coordinators: Kevin Daniels, Melanie Turner

Loma Linda University Health Care, Loma Linda, CA: Principal investigator: Jeffrey Rosenfeld; Sub-investigator: Laura Nist; Study coordinators: Julie Calleros-Lacanlale, Blanca Herrera, Imran Qasim, David Borg, Josie Sedano, Sheba Baroya

Medical University of South Carolina, Charleston, SC: Principal investigator: Katherine Ruzhansky; Sub-investigator: Kimberly Robeson-Gewuerz; Study coordinator: Aparna Choudhury

Michigan State University, East Lansing, MI: Principal investigator: Amit Sachdev; Sub-investigators: Rachel Rosenbaum, Kabelo Thusang; Study coordinators: Kimberly Patterson, Doozie Russell, Aubrey Alexander

MS and Neuromuscular Center of Excellence, Clearwater, FL: Principal investigator: Jean-Raphael Schneider; Sub-investigators: Stuart Sinoff, Dawn Rush Wilde; Study coordinator: Linda Seibert

Neuromuscular Research Center, Phoenix, AZ: Principal investigator: Kumaraswamy Sivakumar; Sub-investigators: Preethi Nimalka Sivakumar, Sidney Spector, Deborah Taylor; Study coordinator: Kristy Osgood

Neurosciences Institute, Neurology-Charlotte, Charlotte, NC: Principal investigator: Urvi Desai; Sub-investigators: Benjamin Brooks, Dina Krystine Forero, Scott Holsten, Cynthia Lary, Leo McCluskey Allison Newell-Sturdivant, Amber Ward; Study coordinators: Jennifer Mabry, Candace Roberson

Northside Hospital, Atlanta, GA: Principal investigator: Gavin Brown; Sub-investigators: Elizabeth Boyter, Roland Hamilton; Study coordinators: Nancy Bryant, Jade Cunningham, Christopher Davis

Northwell Health Physicians Partners Neuroscience Institute, Great Neck, NY (previously Northwell Health Neuroscience Institute at Great Neck)**:** Principal investigator: Anthony P. Geraci; Sub-investigator: Sami Saba; Study coordinators: Scott Baron, Rashmi Kanagaratnam

Northwell Health, New York, NY: Principal investigator: Sami Saba; Sub-investigator: Anthony Geraci; Study coordinators: Scott Baron, Martha Karran

Northwest Neurology, Ltd. Clinical Research, Rolling Meadows, IL: Principal investigator: Andrew J. Gordon; Sub-investigators: Ahmir Khan, Karen Frizelis; Study coordinator: Maureen Ganser

Northwestern University, Feinberg School of Medicine, Department of Neurology, Chicago, IL: Principal investigator: Senda Ajroud-Driss; Sub-investigator: Robert Sufit; Study coordinator: Benjamin Joslin

OhioHealth Neurological Physicians, Columbus, OH: Principal investigator: Timothy Rust; Sub-investigators: Fatimata Diallo, Geoffrey Eubank, Craig Zeid; Study coordinators: Craig Zeid (Primary), Morgan Davis, Fatimata Diallo, Abdul Sadio

Olive View UCLA Medical Center, Sylmar, CA: Principal investigator: Shri Kant Mishra; Sub-investigators: Frank Diaz, Robert Freundlich; Study coordinators: Brooke Benavides, Shaweta Khosa

Oregon Neurology, Springfield, OR: Principal investigator: David Clark; Sub-investigators: Brittany Farro, Jody Nichols, Kristin Tsue; Study coordinators: Jody Nichols, Kristin Tsue

Penn Medicine University City, Philadelphia, PA: Principal investigator: Sami L. Khella; Sub-investigators: Maria Fang Chun Chen, Aron Schwartz; Study coordinator: Aron Schwartz

Penn State Health Milton S. Hershey Medical Center, Hershey, PA: Principal investigator: Sankar Bandyopadhyay; Sub-investigator: Divpreet Kaur; Lead study coordinator: Doris Dodi Schaak; Back-up study coordinator: Yojana Kanade

Spectrum Health, Grand Rapids, MI: Principal investigator: Paul Twydell; Sub-investigator: Matthew Ebright; Study coordinators: Jessica Gallavin, Ryan Groseclose, Laura Holman, Sehrish Khan, Marlie Lieberman, Julia Reddy, Kate Root, Heidi Taylor

St. Joseph's Hospital and Medical Center, Phoenix, AZ: Principal investigator: Suraj A. Muley; Sub-investigators: Jacquelyn Daniels, Bill Jacobsen, Erick Ortega; Study coordinators: Gale Kittle, Lisa Koch, Cassie Nelson

Stanford Neurosciences Health Center, Stanford, CA: Principal coordinator: Srikanth Muppidi; Sub-investigators: John Day, Neelam Goyal, Sarada Sakamuri, Jacinda Sampson, Yuen Tat So, Connie Wolford; Study coordinators: Tia Nguyen, Mitchell Reddan, Veronica Stevens

Stony Brook Clinical Research Center, East Setauket, NY: Principal investigator: Rahman Pourmand; Sub-investigators: Jessica Lamb, Simona Treidler; Study coordinators: Diana Kaell, Jessica Lamb

Swedish Neuroscience Institute, Seattle, WA: Principal investigator: Kristyn Pocock; Sub-investigator: Michael Elliot; Study coordinators: Caryl Tongco, Jen Cardey, Lindsey Maassel

Texas Neurology, P.A., Dallas, TX: Principal investigator: Alan W. Martin; Sub-investigators: Mohamad Asaad Nasri, Maria Phillip; Study coordinators: Yvonne Berry, Kate Cruickshank, Todd Morgan

The Ohio State University Wexner Medical Center, Columbus, OH: Principal investigator: Bakri H. Elsheikh; Sub-investigator: Miriam L. Freimer, Sarah Heintzman; Study coordinators: Keirsten Adkins, Julie Agriesti

The University of North Carolina, Chapel Hill, NC: Principal investigator: James F. Howard Jr; Previous principal investigator: Rebecca Traub; Sub-investigator: Rebecca Traub; Study coordinator: Manisha Chopra

Tufts Medical Center, Boston, MA: Principal investigator: Mithila Vullaganti; Sub-investigators: Taha Bali, Paula Dabenigno, Pradnya Ghule, Emma Jost-Price; Previous sub-investigator: David Thaler; Study coordinators: Leidy Guzman, Keri Sullivan

University Hospitals Cleveland Medical Center, Cleveland, OH: Principal investigator: Bashar Katirji; Sub-investigators: Natalie Abraham, Mary Andrews, Elisar Khawam, David Korosec, Komal Sawlani; Study coordinators: Elisar Khawam, David Korosec

University of California, Irvine, CA: Principal investigator: Ali A. Habib; Sub-investigators: Jonathan Cauchi, Tahseen Mozaffar; Study coordinator: Isela Hernandez

University of Chicago, Chicago, IL; Principal investigator: Betty C. Soliven; Sub-investigator: Kourosh Rezania; Study coordinator: Usman Alvi

University of Cincinnati Gardner Neuroscience Institute, Cincinnati, OH: Principal investigator: Hani Kushlaf; Sub-investigator: Robert Neel; Study coordinator: Sharon Briley

University of Colorado Hospital, Aurora, CO: Principal investigator: Stacy Dixon; Sub-investigators: Dianna Quan, Debra O'Reilly; Primary study coordinator: Emily Hyslop; Study coordinators: Brianna Blume, Kimberley Clawson-Stone

University of Florida Jacksonville, Jacksonville, FL (previously University of Florida Health Science Center)**:** Principal investigator: Michael T. Pulley; Sub-investigators: Alan Berger, Shannon LaBoy, Jeffrey Shije; Study coordinators: Zubair Quraishi, Yasmeen Shabbir

University of Kansas Medical Center, Kansas City, KS: Principal investigator: Mazen M. Dimachkie; Sub-investigators: Richard J. Barohn, Constantine Farmakidis, Duaa Jabari, Omar Jawdat, Mamatha Pasnoor, Jeffrey Statland; Study coordinators: Samantha Colgan, Andrew Heim, Melissa Hayes, Katie Jennens Sandhya Sasidharan

University of Kentucky Chandler Medical Center, Lexington, KY: Principal investigator: Zabeen K. Mahuwala; Sub-investigators: Kyle Darpel, Ima Ebong, Stephen Ryan, Monica Santa-Teresa, Padmaja Sudhakar; Rani Priyanka Vasireddy, Renee Wagner; Study coordinators: Rani Priyanka Vasireddy, Renee Wagner

University of Missouri School of Medicine, Columbia, MO: Principal investigator: Syed Shah; Previous principal investigator: Raghav Govindarajan; Sub-investigators: Andrea Atkins, Amer Avdagic, Stacia Reilly, Natalie Taylor, LiYan Yin; Study coordinator: Karim Salame

University of Pennsylvania, Pennsylvania, PA: Principal investigator: Shawn Bird; Sub-investigator: Christyn Edmundson; Study coordinator: Kelsey Moulton

University of South Florida, Tampa, FL: Principal investigator: Tuan H. Vu; Sub-investigators: Jerrica Farias, Niraja Suresh; Study coordinators: Naraly Requena, Brittany Harvey, Jessica Shaw, Erik Velasquez

University of Texas Health Science Center at San Antonio, San Antonio, TX: Principal investigator: Ratna Bhavaraju-Sanka; Sub-investigators: Valerie Armstrong, Carlayne Jackson; Study coordinators: Floyd Jones, Wesley Lowell, Charlotte Rhodes

University of Texas Health Science Center at Houston, Houston, TX: Principal investigator: Thy Nguyen; Sub-investigators: Suur Biliciler; Study coordinators/Study nurses: Estela Acosta, Carla Wilkerson, Michelle Mayon

University of Vermont, Burlington, VT: Principal investigator: Rup Tandan; Sub-investigator: Waqar Waheed; Study coordinators: Avery St. Sauveur, Lisa Smith, Shannon Lucy

Vanderbilt University Medical Center, Nashville, TN: Principal investigator: Peter D. Donofrio; Sub-investigator: Diana Davis; Study coordinator: Diana Davis

Virginia Commonwealth University, Richmond, VA: Principal investigator: Kelly Gwathmey; Sub-investigator: Nicholas Johnson; Study coordinators: Kelly Huckstep, Bridget Wilkins

Wake Research Clinical Research Center of Nevada, LLC, Las Vegas, NV: Principal investigator: Simon J. Farrow; Sub-investigators: Robert Balsiger, Paul Janda, Jay Mahajan; Primary study coordinator: Amy Kill; Back-up study coordinators: Sierra Dansbee, Bernie Kompancaril, Darlene Steljes; Regulatory study coordinator: Shonda Lester; Study coordinator: Adolfo DeLeon

Washington University School of Medicine, St. Louis, MO: Principal investigator: Muhammed Al Lozi; Sub-investigators: William Buell, Julaine Florence, Gurpreet Khakh, Stephanie Poelker, June Smith, Abigail Yenzer; Study coordinator: June Smith

Wayne State University School of Medicine, Detroit, MI: Principal investigator: Robert P. Lisak; Sub-investigators: Evanthia Bernitsas, Jacob Rube; Study coordinator: Kelly Jia

Wesley Neurology Clinic, Cordova, TN: Principal investigator: Tulio E. Bertorini; Sub-investigators: Cindy Benzel, Yaohui Chai; Robert Henegar, Pheadra Ruffin; Study coordinators: Cindy Benzel, Robert Henegar, Amanda Huffman

West Virginia University, Morgantown, WV: Principal investigator: Cheryl Smith; Sub-investigators: Gauri V. Pawar, Shumaila Sultan; Study coordinators: Patricia Altemus, Lauren Chase, Amanda Lavery

Yale New Haven Hospital, Yale University, New Haven, CT: Principal investigator: Richard J. Nowak; Sub-investigator: Bhaskar Roy; Study coordinators: Skye Gallagher, Joan Nye, Bailey Sheldon

Table S1. Summary of deaths in the CHAMPION MG study

Patient (cause of death)	Age	Sex	Comorbidity[a]	Comedication status	Vaccination status	Duration of ravulizumab treatment[b]	Timing of death relative to last dose of ravulizumab[b]
**1 (Cerebral hemorrhage)**	72 yr	Male	• Abdominal discomfort and shoulder operation<br>• Atrial fibrillation<br>• Constipation<br>• Diabetes mellitus<br>• Hyperlipidaemia<br>• Hypertension<br>• Iron deficiency anemia<br>• Shoulder operation<br>• Sinusitis<br>• Benign prostatic hyperplasia<br>• Lipoma<br>• Nerve root injury cervical<br>• Osteoporosis	• Atorvastatin<br>• Edoxaban tosilate<br>• Ferrous sodium citrate<br>• Fexofenadine hydrochloride<br>• Lansoprazole<br>• Losartan potassium<br>• Lubiprostone<br>• Paracetamol/tramadol hydrochloride<br>• Sitagliptin phosphate<br>• Ciclosporin<br>• Prednisolone<br>• Pyridostigmine bromide<br>• Minondronic acid	IM meningococcal vaccine ACYW	127 days	18 days
**2 (COVID-19 infection)**	72 yr	Male	• Atrial septal defect<br>• Obesity<br>• Hyperlipidemia<br>• Hypertension<br>• Lipoma<br>• Pollakiuria<br>• Disseminated varicella zoster virus infection<br>• Pickwickian syndrome	• Erythromycin<br>• Lisinopril<br>• Simvastatin<br>• Tamsulosin<br>• Mycophenolate mofetil<br>• Prednisone<br>• Pyridostigmine	Meningococcal vaccine B and meningococcal vaccine ACYW	184 days	32 days

Patient (cause of death)	Age	Sex	Comorbidity[a]	Comedication status	Vaccination status	Duration of ravulizumab treatment[b]	Timing of death relative to last dose of ravulizumab[b]
			• Sleep apnea syndrome<br>• Tinnitus<br>• Visual impairment<br>• Glucose tolerance impaired<br>• Hematuria<br>• Breast pain<br>• Rash				
**3 (COVID-19 infection)**	54 yr	Male	• Seasonal allergy<br>• Blood cholesterol increased<br>• Sleep apnea syndrome<br>• Hypertension<br>• Anxiety<br>• Atrial fibrillation<br>• Aortic arteriosclerosis<br>• Hyperlipidemia<br>• Edema peripheral	• Acetylsalicylic acid<br>• Alprazolam<br>• Apixaban<br>• Atorvastatin<br>• Benzonatate<br>• Calcium<br>• Codeine phosphate<br>• Guaifenesin<br>• Colecalciferol<br>• Diltiazem hydrochloride<br>• Fluticasone propionate<br>• Furosemide<br>• Loratadine<br>• Lolmesartan<br>• Salbutamol sulfate<br>• Vitamin B12	Meningococcal vaccine B and Meningococcal vaccine ACYW	127 days	69 days

Patient (cause of death)	Age	Sex	Comorbidity[a]	Comedication status	Vaccination status	Duration of ravulizumab treatment[b]	Timing of death relative to last dose of ravulizumab[b]
**4** **(COVID-19 pneumonia)**	59 yr	Male	• Cholelithiasis<br>• Diverticulum<br>• Hepatic steatosis<br>• Steroid diabetes<br>• Dyspnea<br>• Cough<br>• Hyperglycemia<br>• Obesity	• Calcium/colecalciferol<br>• Colecalciferol<br>• Omeprazole<br>• Potassium chloride<br>• Prednisone<br>• Pyridostigmine	Meningococcal vaccine ACWY and meningococcal vaccine B	127 days	52 days

[a]Ongoing and status unknown conditions only; [b]Time from first ravulizumab infusion to date of last ravulizumab infusion
IM, intramuscular; yr, years
